# Gaps in diversity and inclusion reporting in United States knee injury clinical trials: A systematic review and meta‐analysis

**DOI:** 10.1002/jeo2.70255

**Published:** 2025-05-12

**Authors:** Faith Hendrickson, Caleb Uhunmwangho, Christian Hemmerich, Garrett Jones, J. Tyler Babek, Haley Howard, Jake Checketts, Alicia Ito Ford, Matt Vassar

**Affiliations:** ^1^ Office of Medical Student Research Oklahoma State University Center for Health Sciences Tulsa Oklahoma USA; ^2^ Department of Orthopedic Surgery Oklahoma State University Center for Health Sciences Tulsa Oklahoma USA; ^3^ Department of Psychiatry and Behavioral Sciences Oklahoma State University Center for Health Sciences Tulsa Oklahoma USA

**Keywords:** Clinical Trial Diversity, health disparities, knee, meta‐analysis

## Abstract

**Purpose:**

Diversity and inclusion in clinical trials are critical for increasing the generalizability of research. Our objective was to examine the diversity and inclusion of historically marginalized populations in clinical trials focused on knee injuries in the United States.

**Methods:**

Our systematic review and meta‐analysis evaluated the diversity and representation of clinical trials concerning knee injuries published between 2018 and 2023. Published manuscripts of relevant knee injury clinical trials were identified using the medical literature databases MEDLINE (PubMed) and Embase (Elsevier). Two masked authors independently completed data screening and extraction. We evaluated studies using the Clinical trial Diversity Rating framework to assess their inclusion across multiple demographic characteristics.

**Results:**

A total of 13 studies met the inclusion criteria for the final meta‐analysis. Only 1 out of 13 (7.7%) received a ‘Fair’ representation score for race/ethnicity participation, and 1 out of 13 (7.7%) received a ‘Poor’ representation score. The remaining 11 out of 13 (84.6%) studies did not report information on the race/ethnicity of their participants. Eight out of 13 (61.5%) trials received a ‘Good’ representation score when evaluating the inclusion of males and females, 3/13 (23.1%) were ‘Fair’ and 2/13 (15.4%) were ‘Poor’. None of the studies reported the number of participants aged ≥65 years.

**Conclusion:**

The results of this study highlight a lack of demographic reporting in knee injury clinical trials, with the included studies consistently failing to report information about the race/ethnicity and age breakdown of participants. The lack of diversity goals and insufficient reporting of racial and ethnic minority populations underscores the necessity for strategic approaches going forward to ensure clinical trials are more inclusive and representative of the incidence of knee injuries in the population.

**Level of Evidence:**

Level III, systematic review and meta‐analysis.

**Abbreviations**
CDR: Clinical trial Diversity Rating
OSF: Open Science Framework
PDRR: participation‐to‐disease representation ratio
PRISMA: Preferred Reporting Items for Systematic reviews and Meta‐Analyses

## INTRODUCTION

Knee injuries, including anterior cruciate ligament tears [6], meniscus tears and other ligamentous and cartilage injuries, are common musculoskeletal conditions that affect a broad spectrum of the population [2]. These injuries can result from a variety of activities ranging from high‐impact sports to everyday movements, and can significantly impair mobility, quality of life and overall function [2, 27]. As such, these injuries are a critical area of focus in clinical research aimed at developing effective treatments and rehabilitation protocols.

The generalizability of clinical research heavily depends on the diversity of trial participants [25]. It is well recognized that race/ethnicity, sex and age can influence both the incidence of knee injuries and the outcomes of their treatment [6]. For instance, physiological differences across demographic groups can affect injury susceptibility, healing rates and response to interventions [6, 33]. Despite these differences, there has historically been a lack of reporting of participant diversity in orthopaedic clinical trials, leading to findings that may not be universally applicable [25].

This study evaluated the representation of race/ethnicity, sex, and age in published manuscripts of clinical trials for knee injuries. Using the Clinical trial Diversity Rating (CDR) framework developed by Agboola and Wright, we aimed to assess how well the demographic composition of trial participants reflects the proportions of these groups in the knee injury patient population [1].

Through this meta‐analysis, we sought to identify potential disparities in trial participation and advocate for more inclusive research practices. This is particularly important for knee injuries, which affect individuals across a wide range of ages, races and activity levels, necessitating tailored and effective treatment approaches for diverse patient groups. Our objective was to examine the diversity and inclusion of historically marginalized populations in published manuscripts of clinical trials focused on knee injuries in the United States. We hypothesized that there would be deficiencies in the reporting of clinical trial participant information and a lack of diversity in clinical trial participants.

## METHODS

### Study design

Our systematic review and meta‐analysis evaluated the diversity and representation of knee injury clinical trials using the CDR framework previously developed by Agboola and Wright [1]. We sought to ensure study accuracy and reproducibility by following the Preferred Reporting Items for Systematic reviews and Meta‐Analyses (PRISMA) 2020 checklist [19]. To promote transparency and accessibility, our methods were made publicly available on the Open Science Framework (OSF) [18].

### Search strategy

To aid in the development of our search string, the Cochrane Database of Systematic Reviews of Interventions was searched for knee injury‐related reviews. MeSH terms commonly used in relevant articles were compiled, for example, ‘anterior cruciate ligament’ [MeSH Terms], ‘meniscus’ [MeSH Terms] and ‘patella’ [MeSH Terms]. The complete search string is available on OSF [18]. A systematic literature review was conducted on 28 May 2024—using the medical literature databases MEDLINE (PubMed) and Embase (Elsevier) to identify published manuscripts of clinical trials on knee injuries and associated treatments. Following the search, returns from each medical literature database were uploaded to the systematic review screening platform Rayyan [21]. Following deduplication, two authors (FH AND CU) screened the titles and abstracts in a masked duplicate fashion according to Cochrane guidelines [5].

### Eligibility criteria

To qualify for inclusion, each clinical trial met the following criteria: (1) published or available on 1 January 2018 through 31 December 2023, (2) assessed the effects of an intervention on enrolled participants with knee injuries (anterior cruciate ligament, posterior cruciate ligament, medial collateral ligament, lateral collateral ligament, meniscal or patellar injury) and (3) was conducted in the United States. The following studies were excluded: published outside of the date range, studies not concerning knee injuries, secondary analyses, interim analyses, erratum, corrigendum, trials with international sites, trial updates, protocols, abstracts, studies in foreign languages, animal studies and cadaver studies. After independent screening of all search returns for inclusion eligibility, the two screening authors (FH and CU) reconciled their decisions. If a consensus was not met, a third author (CH) was available for reconciliation.

### Data extraction

Data extraction was conducted independently in a duplicate, masked manner by two authors (FH and CU) using a pilot‐tested Google Form. The following general study data were recorded: year of publication, title, trial site type (single‐centre or multi‐centre), trial phase, funding source, total sample size, blinding and type of intervention (behavioural, pharmaceutical, surgical/procedural or supplement/over the counter). For demographic data, we collected available information on age, sex, race and ethnicity of participants. These demographics were chosen because they are essential for assessing representation per Agboola and Wright [1]. For the following demographics, we included reporting status (Y/N), and the percentage of participants identified as: American Indian/Alaska Native, Asian, Black, Hawaiian/Pacific Islander, white, Hispanic/Latinx, and male and female. Age data included reporting status (Y/N), age ranges, mean with standard deviation and median with interquartile range as reported by the study. When necessary, a search using the National Clinical Trial number was conducted to find additional study data for race, ethnicity, sex and age of participants if this information was not included in the manuscript. After independently completing the extractions, two authors (FH and CU) reconciled their decisions. If a consensus could not be reached, a third author (CH) was available.

### Statistical analysis

#### Participation‐to‐disease representation ratio (PDRR) score and descriptive statistics

We evaluated the representation of age, race/ethnicity, and sex in our sample of clinical trials relative to their proportions in the disease population. The PDRR measures a specific subgroup's participation in a clinical trial compared to their representation in the disease population and is the foundation for the CDR framework. Due to knee injuries not being a disease state, we use incidence data instead of prevalence. Incidence data for sex and age were sourced from a peer‐reviewed article [11]. The US Census data was used to define race and ethnicity incidence distributions, due to specific race incidence data for knee injury not being readily available [30]. PDRR was calculated by taking the percentage of study participants from a specific subgroup and dividing it by the incidence of a knee injury within that same subgroup (or, for race/ethnicity, the % of the population based on the US census). For example, if a condition affects females in 50% of cases, and a clinical trial regarding this condition includes 20% female subjects, the PDRR would be 0.4 (0.20/0.50). Previous studies have defined a PDRR between 0.8 and 1.2 as adequate representation. Additionally, a PDRR < 0.8 suggests underrepresentation, and a PDRR > 1.2 suggests overrepresentation [1, 7, 20]. We adopted these scoring criteria in our analysis. The CDR framework then assigns a score (0–3) for each demographic category, based upon the PDRR value, using the following thresholds: 3 for PDRR ≥ 0.8, 2 for PDRR ≥ 0.5 to <0.8, 1 for PDRR > 0 to <0.5, and 0 for PDRR of 0 [1]. Because race and ethnicity are standard reporting items for US studies, unreported values were calculated as 0. These scores are then summed up based on the overall demographic characteristics and assigned an overall diversity rating of ‘Good’, ‘Fair’ or ‘Poor’.

#### Meta‐analysis and subgroup analysis

We used meta‐analytic techniques to characterize participant diversity within knee injury clinical studies. For calculations, the PDRR score operated as the point estimate to quantify the relative representation of demographic groups between studies. Standard error was calculated using the individual study sample size as *n*. The upper and lower confidence intervals were first calculated on the log scale using the natural log‐transformed PDRR and the standard error. The log transform accounted for the skewed distributions commonly found when analyzing ratios. The confidence intervals were then back‐transformed by taking the exponential of the upper and lower confidence intervals. The final PDRR and upper and lower 95% confidence intervals were then used to create the forest plots to illustrate the data.

## RESULTS

### PRISMA diagram

**Figure 1:**
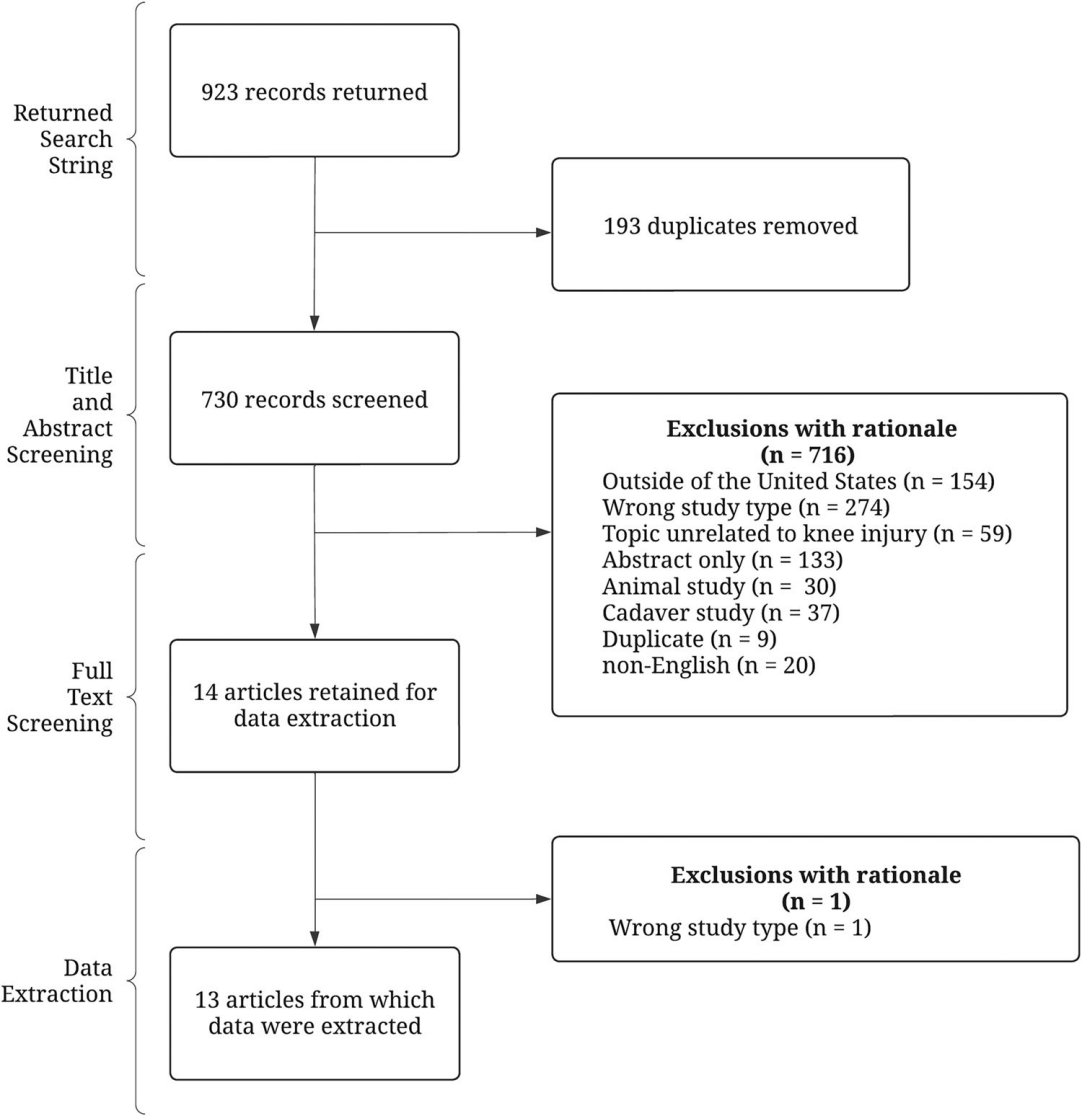
Flow diagram of study selection.

Our systematic literature search yielded 923 records. After deduplication, 730 records were screened by title and abstract, resulting in the exclusion of 716 records. We then conducted a full‐text review of 14 articles. Based on our screening process and inclusion criteria, 13 studies were included in the final analysis [3, 9, 10, 12, 13, 15, 17, 24, 26, 28, 31, 32, 35]. A list of the included studies is publicly available on OSF [18]. The reasons for exclusion are detailed in Figure 1.

### General characteristics

The majority of studies were conducted at a single‐centre trial site (11/13, 84.6%) and involved a surgical or procedural intervention (9/13, 69.2%). Almost half of the studies had a sample size of <50 participants (6/13, 46.2%) and were conducted in a single‐blind manner (6/13, 46.2%). Additionally, most studies (11/13, 84.6%) were inapplicable for a clinical trial phase. Further characteristics are presented in Table 1.

**Table 1:** Summary of characteristics of included studies (*n* = 13).

Review characteristics	No. (%)
Sample size	
<50	6 (46.2%)
50–100	2 (15.4%)
101–150	5 (38.5%)
>150	0 (0%)
No. of centres conducting clinical trial	
Single‐centre	11 (84.6%)
Multi‐centre	2 (15.4%)
Funding source	
No funding statement provided	5 (38.5%)
Private	0 (0%)
Industry	2 (15.4%)
No funding received	0 (0%)
Government	2 (15.4%)
Hospital/university	1 (7.7%)
Self‐funded	0 (0%)
Multiple	3 (23.1%)
Type of intervention	
Pharmaceutical	1 (7.7%)
Behavioural	2 (15.4%)
Supplemental/OTC	1 (7.7%)
Surgical/procedural	9 (69.2%)
Other	0 (%)
Clinical trial phase	
Phase 1	0 (%)
Phase 2	0 (%)
Phase 3	1 (7.7%)
Phase 4	1 (7.7%)
Not applicable	11 (84.6%)
Blinding	
Single	6 (46.2%)
Double	4 (30.8%)
Triple	0 (0%)
None	3 (23.1%)

Abbreviation: OTC, over the counter.

Table 2 presents the knee injury incidence estimates, proportion of participation by sex, older adults (≥65 years of age), race/ethnicity and the diversity rating scores. Evaluating the overall representation of males and females, 8 out of 13 (61.5%) trials received a ‘Good’ representation score, 3 out of 13 (23.1%) received a ‘Fair’ score and 2 out of 13 (15.4%) received a rating of ‘Poor’. One out of 13 (7.7%) received a ‘Fair’ representation score for race/ethnicity participation, and 12 out of 13 (92.3%) received a ‘Poor’ representation score. Of the 12 studies with a ‘Poor’ representation score, 11 out of 13 (84.6%) did not report information on the race/ethnicity of participants. None of the studies (0/13) reported the number of participants included ≥65 years of age, so no diversity ratings could be calculated for the age subgroups.

**Table 2:** Detailed use of the CDR framework for the included studies.

	Incidence of knee injuries in the US	% of participants	Participant to disease‐representation ratio	Representation score (score ranges from 0 to 3)	Total representation score	Diversity rating
Capin et al. [3] (*N* = 40)						
Sex					0	POOR
Female (*n* = 40)	47.78%	100.00%	2.09	3		
Male (*n* = 0)	52.22%	0.00%	0.00	0		
Age					–	–
Older adults (*n* = NR)	27.84%	NR	NC	NC		
Race/ethnicity					0	POOR
White (*n* = NR)	58.90%	NR	0.00	0		
Black (*n* = NR)	13.60%	NR	0.00	0		
Asian (*n* = NR)	6.30%	NR	0.00	0		
Hispanic (*n* = NR)	19.10%	NR	0.00	0		
Fleming et al. [9] (*N* = 150)						
Sex					6	GOOD
Female (*n* = 74)	47.78%	49.33%	1.03	3		
Male (*n* = 76)	52.22%	50.67%	0.97	3		
Age					–	–
Older adults (*n* = NR)	27.84%	NR	NC	NC		
Race/ethnicity					0	POOR
White (*n *= NR)	58.90%	NR	0.00	0		
Black (*n* = NR)	13.60%	NR	0.00	0		
Asian (*n* = NR)	6.30%	NR	0.00	0		
Hispanic (*n* = NR)	19.10%	NR	0.00	0		
Forsythe et al. [10] (*N* = 81)						
Sex					5	FAIR
Female (*n* = 48)	47.78%	59.26%	1.24	3		
Male (*n* = 33)	52.22%	40.74%	0.78	2		
Age					–	–
Older adults (*n* = NR)	27.84%	NR	NC	NC		
Race/ethnicity					0	POOR
White (*n* = NR)	58.90%	NR	0.00	0		
Black (*n* = NR)	13.60%	NR	0.00	0		
Asian (*n* = NR)	6.30%	NR	0.00	0		
Hispanic (*n* = NR)	19.10%	NR	0.00	0		
Hartwell et al. [12] (*N* = 95)						
Sex					6	GOOD
Female (*n* = 44)	47.78%	46.32%	0.97	3		
Male (*n* = 51)	52.22%	53.68%	1.03	3		
Age					–	–
Older adults (*n* = NR)	27.84%	NR	NC	NC		
Race/ethnicity					0	POOR
White (*n* = NR)	58.90%	NR	0.00	0		
Black (*n* = NR)	13.60%	NR	0.00	0		
Asian (*n* = NR)	6.30%	NR	0.00	0		
Hispanic (*n* = NR)	19.10%	NR	0.00	0		
Hogan et al. [13] (*N* = 119)						
Sex					6	GOOD
Female (*n* = 50)	47.78%	42.02%	0.88	3		
Male (*n* = 69)	52.22%	57.98%	1.11	3		
Age					–	–
Older adults (*n* = NR)	27.84%	NR	NC	NC		
Race/ethnicity					0	POOR
White (*n* = 93)	58.90%	78.15%	1.33	3		
Black (*n* = NR)	13.60%	NR	0.00	0		
Asian (*n* = NR)	6.30%	NR	0.00	0		
Hispanic (*n* = NR)	19.10%	NR	0.00	0		
Krych et al. [15] (*N* = 45)						
Sex					5	FAIR
Female (*n* = 29)	47.78%	65.44%	1.37	3		
Male (*n* = 16)	52.22%	35.56%	0.68	2		
Age					–	–
Older adults (*n* = NR)	27.84%	NR	NC	NC		
Race/ethnicity					0	POOR
White (*n* = NR)	58.90%	NR	0.00			
Black (*n* = NR)	13.60%	NR	0.00			
Asian (*n* = NR)	6.30%	NR	0.00			
Hispanic (*n* = NR)	19.10%	NR	0.00			
Murray et al. [17] (*N* = 100)						
Sex					6	GOOD
Female (*n* = 56)	47.78%	56.00%	1.17	3		
Male (*n* = 44)	52.22%	44.00%	0.84	3		
Age						–
Older adults (*n* = NR)	27.84%	NR	NC	NC		
Race/ethnicity					8	FAIR
White (*n* = 83)	58.90%	83.00%	1.41	3		
Black (*n* = 2)	13.60%	2.00%	0.15	1		
Asian (*n* = 12)	6.30%	12.00%	1.90	3		
Hispanic (*n* = 1)	19.10%	1.00%	0.05	1		
Solomon et al. [24] (*N* = 26)						
Sex					6	GOOD
Female (*n* = 12)	47.78%	46.15%	0.97	3		
Male (*n* = 14)	52.22%	53.85%	1.03	3		
Age					–	–
Older adults (*n* = NR)	27.84%	NR	NC	NC		
Race/ethnicity					0	POOR
White (*n* = NR)	58.90%	NR	0.00	0		
Black (*n* = NR)	13.60%	NR	0.00	0		
Asian (*n* = NR)	6.30%	NR	0.00	0		
Hispanic (*n* = NR)	19.10%	NR	0.00	0		
Tamura et al. [26] (*N* = 7)						
Sex					3	POOR
Female (*n* = 7)	47.78%	100.00%	2.09	3		
Male (*n* = 0)	52.22%	0.00%	0.00	0		
Age					–	–
Older adults (*n* = NR)	27.84%	NR	NC	NC		
Race/ethnicity					0	POOR
White (*n* = NR)	58.90%	NR	0.00	0		
Black (*n* = NR)	13.60%	NR	0.00	0		
Asian (*n* = NR)	6.30%	NR	0.00	0		
Hispanic (*n* = NR)	19.10%	NR	0.00	0		
Trofa et al. [28] (*N* = 30)						
Sex					6	GOOD
Female (*n* = 11)	47.78%	36.67%	0.84	3		
Male (*n* = 19)	52.22%	63.33%	1.21	3		
Age					–	–
Older adults (*n* = NR)	27.84%	NR	NC	NC		
Race/ethnicity					0	POOR
White (*n* = NR)	58.90%	NR	0.00	0		
Black (*n* = NR)	13.60%	NR	0.00	0		
Asian (*n* = NR)	6.30%	NR	0.00	0		
Hispanic (*n* = NR)	19.10%	NR	0.00	0		
Walters et al. [31] (*N* = 50)						
Sex					6	GOOD
Female (*n* = 28)	47.78%	56.00%	1.17	3		
Male (*n* = 22)	52.22%	44.00%	0.84	3		
Age					–	–
Older adults (*n* = NR)	27.84%	NR	NC	NC		
Race/ethnicity					0	POOR
White (*n* = NR)	58.90%	NR	0.00	0		
Black (*n* = NR)	13.60%	NR	0.00	0		
Asian (*n* = NR)	6.30%	NR	0.00	0		
Hispanic (*n* = NR)	19.10%	NR	0.00	0		
Washabaugh et al. [32] (*N* = 15)						
Sex					6	GOOD
Female (*n* = 10)	66.67%	60.00%	0.90	3		
Male (*n* = 5)	33.33%	40.00%	1.20	3		
Age					–	–
Older adults (*n* = NR)	27.84%	NR	NC	NC		
Race/ethnicity					0	POOR
White (*n* = NR)	58.90%	NR	0.00	0		
Black (*n* = NR)	13.60%	NR	0.00	0		
Asian (*n* = NR)	6.30%	NR	0.00	0		
Hispanic (*n* = NR)	19.10%	NR	0.00	0		
Zaslav et al. [35] (*N* = 127)						
Sex					5	FAIR
Female (*n* = 31)	47.78%	24.40%	0.51	2		
Male (*n* = 96)	52.22%	75.60%	1.45	3		
Age					–	–
Older adults (*n *= NR)	27.84%	NR	NC	NC		
Race/ethnicity					0	POOR
White (*n* = NR)	58.90%	NR	0.00	0		
Black (*n* = NR)	13.60%	NR	0.00	0		
Asian (*n* = NR)	6.30%	NR	0.00	0		
Hispanic (*n* = NR)	19.10%	NR	0.00	0		

Abbreviations: CDR, Clinical trial Diversity Rating; NC, not calculable; NR, not reported.

### Meta‐analysis

**Figure 2:**
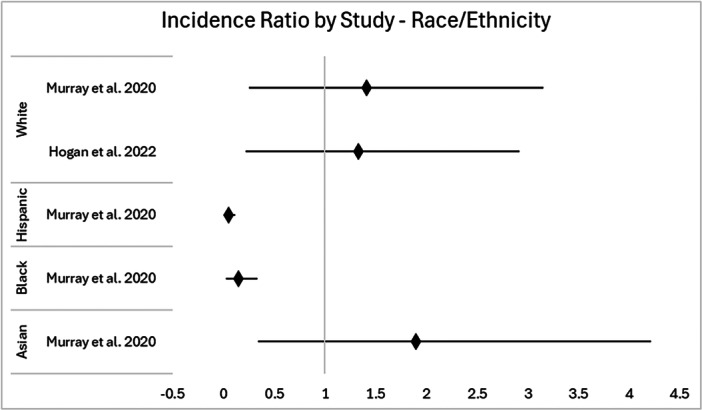
Forest plot of PDRR, with 95% CI (vertical line at one). CI, confidence interval; PDRR, participant‐to‐disease‐representation ratio.

A forest plot of participation ratio for race/ethnicity representation in knee injury clinical trials is displayed in Figure 2.

Two studies reported race and ethnicity data of their participant population (2/13, 15.4%). Of the two, one study specified the white participant proportion but did not report on other race/ethnicity information. In the remaining study, Black and Hispanic/Latinx populations had PDRR values below 0.8, with statistically significant underrepresentation. White and Asian subgroups had PDRR values greater than 1.2, which was considered overrepresentation, though this did not meet statistical significance.

**Figure 3:**
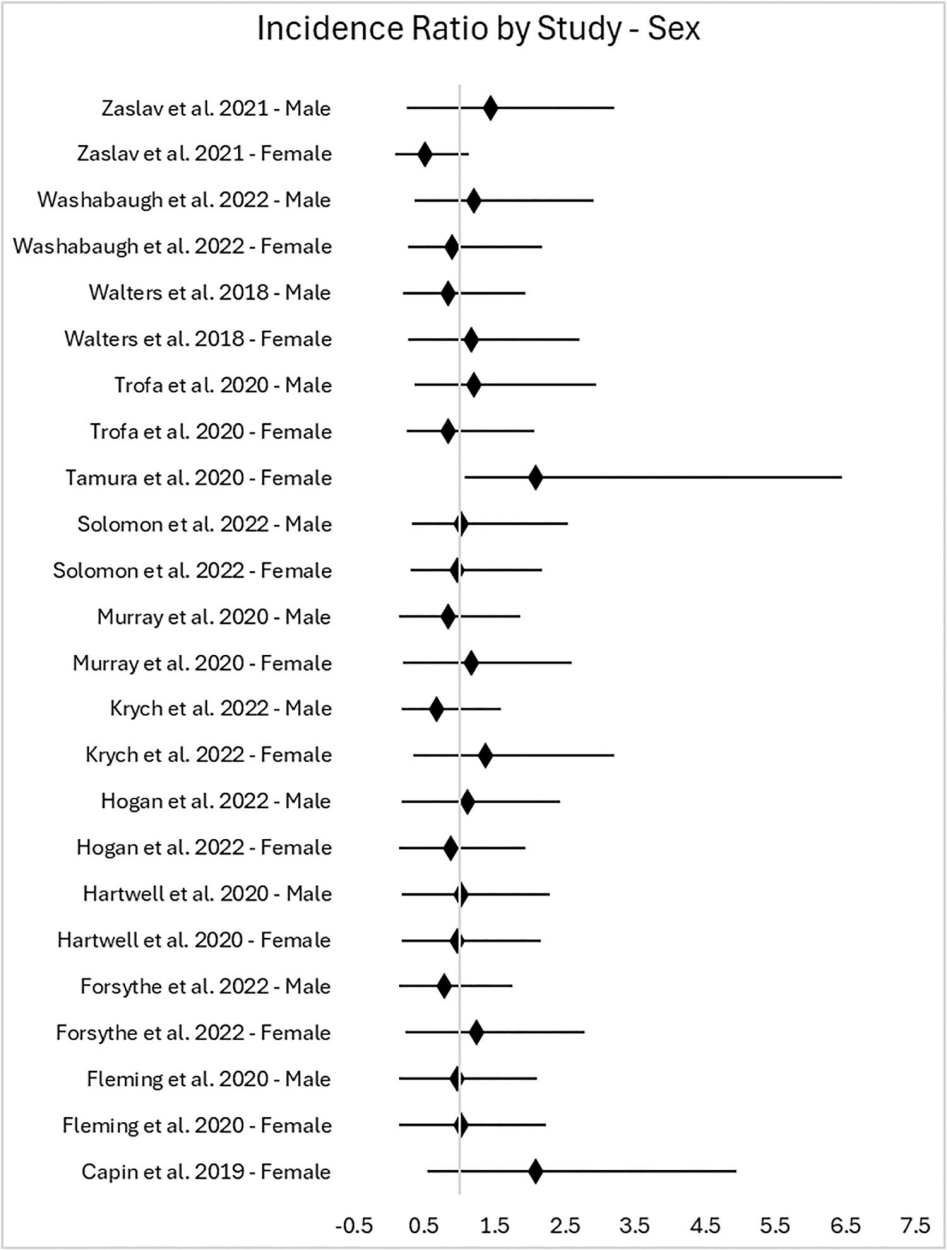
Forest plot of PDRR, with 95% CI (vertical line at one). CI, confidence interval; PDRR, participant‐to‐disease representation ratio.

The participation ratio for females and males in knee injury clinical trials is displayed in Figure 3. Representation in our studies was generally comparable to the knee injury incidence in the United States. Two studies included only female participants. For female participants, almost half of our studies (6/13, 46.2%) had a PDRR above 1.2, which is considered overrepresentation, though the difference was statistically significant in only one study. Adequate male and female representation with a PDRR of 0.8 was present in an equal number of studies (3/13, 23.1%).

## DISCUSSION

Our meta‐analysis revealed that US‐based clinical trials for knee injuries infrequently provide demographic information on the race/ethnicity and age breakdown of study participants. Of the studies analyzed, only a single study reported comprehensive demographic information on the race and ethnicity of trial participants. In this study, white and Asian populations were overrepresented, while Hispanic/Latinx and Black populations were underrepresented relative to the incidence of knee injuries in the general population. The representation scores trended towards ‘Good’, Male/Female representation. Reporting of participant age group breakdown was absent, therefore making it impossible to assess the age representation of the studies. These findings underscore the critical lack of demographic reporting in knee injury clinical trials, highlighting the need for better reporting standards for race and ethnicity and age of study participants.

Our findings align with previous results in orthopaedic and sports medicine literature. Kotlier et al. similarly found that the race and ethnicity of trial participants were reported in less than 10% of randomized controlled trials for shoulder arthroplasty. Furthermore, they found that none of the studies in their sample reported sociodemographic variables in the results section [14]. These results are especially concerning when you factor in that historically marginalized communities have been shown to have unequal health outcomes in the treatment of knee pathologies. For instance, these groups have been reported to have lower rates of knee arthroplasty utilization while experiencing higher rates of adverse health outcomes from the procedure [34]. Health inequities such as this highlight the need for adequate representation in knee injury clinical trials, which can be crucial in identifying causes for inequitable outcomes in knee injury therapies. Furthermore, we found that while every study in our sample provided some statistics regarding the age of participants, it was frequently reported as the mean or median age of participants rather than the number of patients falling in a particular age range. Knee injury therapies and treatment responses are strongly dependent on patient age, as age is an independent risk factor for longer hospital stays, ICU admissions, and complications in some cases [8]. The unique considerations that accompany an individual's age underscore the importance of reporting this information in a standardized manner that allows subsequent analyses.

Despite our results suggesting a lack of diversity in clinical trial participation, we recognize the inherent barriers to recruiting a diverse population in knee injury trials. For example, in traumatic cases, patients with knee injuries will invariably seek care at the closest emergency room. In cases such as this, patients' ability to participate in a clinical trial is dependent on the health system they happen to receive care at and whether that facility is a clinical trial site. Clinical trials with this enrolment model are potentially susceptible to geographical biases, especially if the study has only a few trial sites, as they are limited to patients in the surrounding area. There are cases in which knee injuries are not traumatic or a time sensitive emergency, in which historical barriers such a lack of transportation, financial restraints, fear of adverse effects, and a lack of insurance coverage may be the primary drivers for inadequate representation in clinical trials [23, 25, 29]. Furthermore, barriers can be culture‐specific as seen in communities that report past research abuses as reasons for mistrust in clinical research [22, 25]. Addressing these barriers is crucial for ensuring equitable access to clinical trials, which can lead to more inclusive and generalizable study results, ultimately improving health outcomes for the entire disease population.

A few notable initiatives have highlighted methodology directed towards avoiding the geographic bias of studies being conducted at a limited number of clinical trial sites. One such example is the IRONMAN registry for prostate cancer trials, which has partnered with several consortia to help promote the establishment of geographically diverse trial sites that ensure the recruitment of a diverse sample of participants [16]. Their model includes many international trial sites, such as the African‐Caribbean Cancer Consortium and the Prostate Cancer Transatlantic consortium, to increase opportunities for patients to participate in prostate cancer trials in different populations. In addition to using trials such as this as a template for techniques to overcome geographic biases, we also believe that research journals could have an impact on the use of recruitment strategies. Journals frequently recommend, or require in some instances, the use of reporting guidelines to promote best reporting practices [4]. We believe journals can improve the use of recruitment strategies by requiring clinical trials to report strategies employed in the recruitment of a diverse sample of participants. Together, we believe practices such as these can improve the overall quality of clinical trials by ensuring that trial participants are truly representative of the disease population. The lack of diversity goals and insufficient reporting of racial and ethnic minority populations underscores the necessity for strategic approaches going forward to ensure clinical trials are more inclusive. Ultimately, this will lead to research with increased generalizability, improved understanding of how knee injuries and treatments affect diverse populations, and better recovery outcomes.

### Strengths and limitations

To limit bias, the search results were screened and extracted by two authors (FH and CU) in a duplicate, masked manner following the gold standard defined by Cochrane Collaboration guidelines [5]. Our protocol, extraction forms and data were uploaded to OSF to promote data sharing and transparency [18]. However, we were limited by a reliance on secondary data and the lack of reporting in the studies evaluated. Only 2 out of 13 (15.4%) reported race/ethnicity data, and one of those studies only reported the number of white participants but failed to report the number of Black, Asian, or Hispanic/Latinx participants. Unclear reporting creates uncertainty about the composition of the recruited population, and the study depended on published reports, which may have inconsistencies, reporting biases, or missing data. Our study is limited by a sample size of 13 clinical trials as additional studies could strengthen the correlations. By only including studies conducted in the United States, we may have excluded relevant trials conducted at international sites with different participant inclusion patterns. However, this was intentional due to the difficulty of sourcing reliable demographic and incidence data for each other's country. Additionally, our study is limited by a potential selection bias as the inclusion criteria and search strategy may have inadvertently excluded relevant trials. Our study is also limited by an inability to assess age representation, as no studies reported the number of participants included ≥65 years of age.

## CONCLUSION

The results of this study highlight a lack of demographic reporting in knee injury clinical trials, with the included studies consistently failing to report information about the race/ethnicity and age breakdown of participants. There is a need for knee injury clinical trials to be transparent when reporting demographic information of study participants and to recruit and retain a more diverse population that is representative of the incidence of knee injuries in the population.

## AUTHOR CONTRIBUTIONS

All authors contributed to the study conception and design. Faith Hendrickson completed the data screening and extraction and contributed to the drafting/revision of the article for content, including medical writing for content; analysis or interpretation of data. Caleb Uhunmwangho completed the data screening and extraction and contributed to the drafting/revision of the article for content, including medical writing for content; analysis or interpretation of data. Christian Hemmerich was available as a third author for conflict resolution in the event a consensus could not be reached during data screening/extraction; contributed to the drafting/revision of the article for content, including medical writing for content; analysis or interpretation of data. Garrett Jones contributed to the drafting/revision of the article for content, including medical writing for content; analysis or interpretation of data. J. Tyler Babek contributed to the drafting/revision of the article for content, including medical writing for content; analysis or interpretation of data. Haley Howard contributed to the drafting/revision of the article for content, including medical writing for content; analysis or interpretation of data. Jake Checketts contributed to the drafting/revision of the article for content, including medical writing for content; study concept or design; analysis or interpretation of data. Alicia Ito Ford contributed to the drafting/revision of the article for content, including medical writing for content; study concept or design; analysis or interpretation of data. Matt Vassar contributed to the drafting/revision of the article for content, including medical writing for content; study concept or design; analysis or interpretation of data. All authors read and approved the final manuscript.

## CONFLICT OF INTEREST STATEMENT

Matt Vassar reports receipt of funding from the National Institute on Drug Abuse, the National Institute on Alcohol Abuse and Alcoholism, the U.S. Office of Research Integrity, the Oklahoma Centre for Advancement of Science and Technology, and internal grants from the Oklahoma State University Centre for Health Sciences—all outside of the present work. Alicia Ito Ford reports receipt of funding from the Center for Integrative Research on Childhood Adversity, the Oklahoma Shared Clinical and Translational Resources, and internal grants from Oklahoma State University and the Oklahoma State University Center for Health Sciences—all outside of the present work. The remaining authors declare no conflicts of interest.

## ETHICS STATEMENT

We submitted our study protocol to the institutional review board; it was determined that our study did not include human subjects and required no further supervision.

## Data Availability

The data that support the findings of this study are openly available in the Open Science Framework at https://osf.io/hcd9g/?view_only=19922aa3e0744f609587d45906ee2199.
